# Looking Beyond the Usual Suspects: A Rare Case of Teriparatide-Induced Gynecomastia

**DOI:** 10.1210/jcemcr/luae098

**Published:** 2024-06-26

**Authors:** Bhanvi Ramchandani, Faryal Sardar Mirza

**Affiliations:** Department of Internal Medicine, UConn Health, Farmington, CT 06030, USA; Department of Endocrinology, Diabetes and Metabolism, UConn Health, Farmington, CT 06030, USA

**Keywords:** teriparatide, gynecomastia, osteoporosis, parathyroid hormone, parathyroid hormone receptor, parathyroid hormone-related protein

## Abstract

Teriparatide, an osteoanabolic agent, is a biosynthetic analogue of the 1-34 amino acids of human parathyroid hormone (PTH) used for the treatment of osteoporosis. It is typically well-tolerated; common side effects include headaches, arthralgias, nausea, and dizziness. In this report, we present a case of gynecomastia occurring shortly after initiating teriparatide therapy, associated with nipple sensitivity and breast tenderness. Secondary workup for various causes of gynecomastia was unremarkable. Finally, a decision was made to discontinue teriparatide due to the patient’s concerns. The nipple sensitivity started improving shortly afterward, with complete resolution of gynecomastia 4 months later. Although this unusual side effect has been reported as a possibility in postmarketing studies, a chronological report on the occurrence of teriparatide-induced gynecomastia and its complete resolution after discontinuing teriparatide has not yet been published in the literature.

## Introduction

Osteoporosis is characterized by reduced bone density, increased bone fragility, and a resultant heightened susceptibility to fractures. This poses a significant public health concern and is associated with elevated mortality, impaired quality of life, and increased healthcare costs, particularly in the elderly population.

Teriparatide (TP) is a biosynthetic analogue of the first 34 N-terminal amino acids of parathyroid hormone (PTH), used in the treatment of osteoporosis. It is usually well-tolerated and side effects are usually mild, including nausea, headaches, arthralgias, and dizziness.

We hereby describe an interesting case of gynecomastia that developed shortly after starting treatment with TP, with complete resolution a few months after discontinuation of therapy, suggesting causality.

## Case Presentation

A 61-year-old male physician with a past medical history of gastroesophageal reflux disease (GERD) on chronic proton pump inhibitor (PPI) therapy, hyperlipidemia with statin intolerance, and vitamin D deficiency was found to have a 1.5-inch height loss during his annual physical examination. He was a vegan, and his dietary intake of calcium was suboptimal. He was physically active but denied any recent weight loss. He denied any alcohol, tobacco, or recreational drug use. His medications included 2000 units of vitamin D, ezetimibe, and dexlansoprazole daily. He had never had any fractures and had never taken steroids, chemotherapeutic medications, thyroid medications, seizure medications, or heparin. There was no history of eating disorders. He had been on PPI therapy for 25 years for GERD. He had no history of kidney stones, or chronic inflammatory bowel or lung disease. His family history was significant for ductal carcinoma in situ of the breast and osteoporosis in his mother as well as bilateral hip fractures in his father at the age of 90. He had normal blood pressure on initial examination. Physical examination was essentially unremarkable, except for very mild scoliosis. He did not have any spinal tenderness. Baseline bone mineral density (BMD) was assessed using a Hologic machine, which revealed severe osteoporosis with L1-L4 T-score of −2.6 and BMD of 0.904 g/cm^2^. The left femoral neck mean T-score was −2.9 with BMD of 0.699 g/cm^2^.

He was not eager to take any osteoporosis medications at that time and based on shared decision-making in 2019, he was managed conservatively. He improved his calcium intake to 1000 mg a day (calcium supplement along with almond milk or soy milk) and vitamin D supplementation was increased to 4000 units daily. Over the next year, he was able to gradually taper off dexlansoprazole altogether. Follow-up BMD evaluation 2 years from baseline on a lunar machine showed some worsening of spine BMD (Table 1) based on T-scores. An accurate comparison between the 2 studies could not be done as they were performed at different institutions. The patient was initially hesitant to take any medication and focused on improving his lifestyle and exercise regime while ensuring adequate calcium and vitamin D intake. However, since his bone density continued to decline with relatively low bone turnover markers, he was advised to start anabolic therapy with TP. After some consideration, he was finally agreeable to treatment and was initiated on TP. Four months after starting TP, the patient presented to his primary care physician with bilateral nipple sensitivity and left breast pain without discharge, swelling or redness. He denied having any erectile dysfunction or decline in sexual function.

**Table 1.** DXA scan at the time of diagnosis of osteoporosis, before initiating teriparatide, and 1 year after stopping teriparatide

	Lumbar spine (L1-L4)		Femoral neck		Total hip	
	BMD	T-score	BMD	T-score	BMD	T-score
At initial presentation (Hologic) (2019)	0.880 (g/cm^2^)	−2.8	0.690 (g/cm^2^)	−2.9	0.791 (g/cm^2^)	−2.2
Before initiating TP (GE Lunar Prodigy) (2021)	0.852 (g/cm^2^)	−3.1	0.691 (g/cm^2^)	−2.9	0.813 (g/cm^2^)	−2.0
After stopping TP (GE Lunar Prodigy) (2022)	0.900 (g/cm^2^)	−2.8	0.707 (g/cm^2^)	−2.8	0.803 (g/cm^2^)	−2.1

Abbreviations: BMD, bone mineral density; DXA, dual-energy x-ray absorptiometry; TP, teriparatide.

## Diagnostic Assessment

The patient underwent workup for secondary causes of osteoporosis at initial presentation, which revealed normal serum calcium and creatinine, with a vitamin D level of 28 ng/mL (70 nmol/L) (reference range ≥ 30 ng/mL [≥ 75 nm/L]), despite the patient being on 2000 U of vitamin D. Second fasting morning urine collagen cross-linked N-telopeptide (NTx) was 45 nm bone collagen equivalents (BCE)/mM creatinine (reference range: 21–83 nM BCE/mM creatinine), bone-specific alkaline phosphatase (BSAP) was 9 mcg/L (µg/L) (reference range: 7.6–14.9 mcg/L) and PTH was 37 pg/mL (3.92 pmol/L) (reference range: 14–64 pg/mL), as shown in Table 2. Morning testosterone level was normal. The remainder of the workup was negative for celiac disease and showed normal 24-hour urine calcium. A review of health records through his primary care showed that he had borderline low alkaline phosphatase level a few years prior to presentation, but it had been in the normal range for a couple of years before his initial presentation to us.

**Table 2.** Laboratory markers at the time of diagnosis, before initiating teriparatide, and 5 months after teriparatide therapy (at the time of discontinuation)

Laboratory marker	At initial presentation	Before initiating TP	At the time of stopping TP	Reference range
Creatinine	0.8 mg/dL (70.74 µmol/L)	0.8 mg/dL (70.74 µmol/L)		0.7–1.25 mg/dL (61.9 to 114.9 µmol/L)
GFR	97.8 mL/min/1.73 m^2^	97 mL/min/1.73 m^2^	105 mL/min/1.73 m^2^	>60 mL/min/1.73 m^2^
Calcium	9.2 mg/dL (2.3 mmol/L)	9.7 mg/dL (2.42 mmol/L)	10.3 mg/dL (2.57 mmol/L)	8.6–10.3 mg/dL (2.13 to 2.57 mmol/L)
Albumin	4.9 g/dL (49 g/L)	4.5 g/dL (45 g/L)	4.8 g/dL (48 g/L)	3.8–5.3 g/dL (34 to 54 g/L)
BSAP	9 mcg/L (9 µg/L)	7.8 mcg/L (7.8 µg/L)		7.6–14.9 mcg/L (7.6–14.9 µg/L)
Urine NTx	45 nM BCE/mM creatinine	42 nM BCE/mM creatinine	98 nM BCE/mM creatinine	21–83 nM BCE/mM creatinine
PTH	37 pg/mL (3.92 pmol/L)		78 pg/mL (8.37 pmol/L)	14–64 pg/mL (1.4–6.7 pmol/L)
25(OH)Vit D	30 ng/mL (74 nmol/L)	37 ng/mL (92 nmol/L)	44 ng/mL (109 74 nmol/L)	>30 ng/mL (>74 nmol/L)
Testosterone	476 ng/dL (16 nmol/L)		360 ng/dL (12 nmol/L)	300–720 ng/dL (10–24 nmol/L)

Values in parentheses are International System of Units (SI). Abbreviations: 25(OH)Vit D, 25-hydroxyvitamin D; BSAP, bone-specific alkaline phosphatase; BCE, bone collagen equivalents; PTH, parathyroid hormone; TP, teriparatide; urine NTx, urinary N-telopeptide.

**Figure 1.**
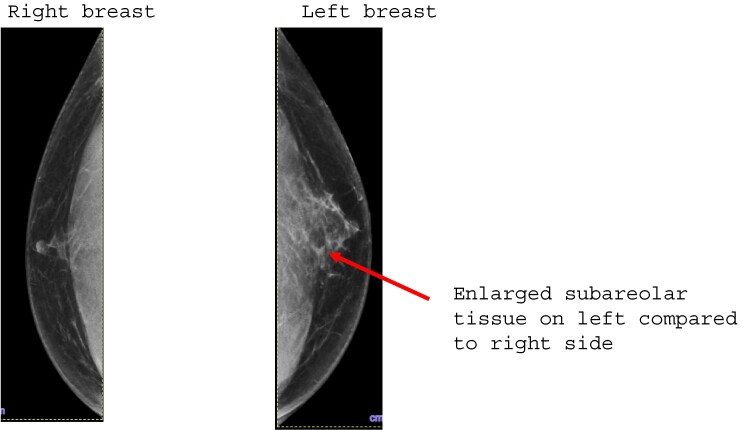
Left and right breast on mammogram. Red arrow showing prominent subareolar tissue under left nipple.

He presented to his primary care physician 4 months after initiating TP with reports of bilateral nipple sensitivity and breast tenderness predominantly on the left side; he was noted to have left nipple tenderness and nodularity in the left breast subareolar tissue on examination. There was no associated bleeding or nipple discharge. Laboratory evaluation for gynecomastia showed normal total and free testosterone level at 360 ng/dL (12 nmol/L) (reference range: 300–720 ng/dL) and 68 pg/mL (2 nmol/L) (reference range: 46–224 ng/dL), respectively, as well as estradiol 39 pg/mL (143 pmol/L) (reference range: 20–47 pg/mL), luteinizing hormone 1.44 mIU/mL (1.4 IU/L) (reference range: 1–11 mIU/mL), follicle-stimulating hormone 4.44 mIU/mL (4.44 IU/L) (reference range: 2.5–9.1 mIU/mL), thyrotropin (thyroid-stimulating hormone) 1.71 uIU/L (1.71 IU/L) (reference range: 0.4–4.5 IU/L), prolactin 4.54 ng/mL (4.54 µg/L) (reference range: 2.64–13.13 µg/L) and human chorionic gonadotropin at < 2 IU/mL (0.2 mIU/mL) (reference range < 5 IU/mL), along with normal creatinine and liver enzymes (see Table 3). Mammogram showed flame-shaped focal asymmetry contiguous with the nipple which was more prominent on the left compared to the right side, compatible with gynecomastia on the left side (Fig. 1). Breast ultrasound showed slightly hypoechoic retro areolar dendritic soft tissue bilaterally, greater on the left compared to the right side, correlating with the mammographic findings.

**Table 3.** Laboratory testing done to evaluate for secondary causes of gynecomastia in our patient

Laboratory parameter	Value	Reference range
Total testosterone	360 ng/dL (12 nmol/L)	300-720 ng/dL (10-24 nmol/L)
Free testosterone	68 ng/dL (2 nmol/L)	46-224 ng/dL (1.5-7 nmol/L)
Estradiol	39 pg/mL (143 pmol/L)	20-47 pg/mL (73-172 pmol/L)
LH	1.4 mIU/mL (1.4 IU/L)	1-11 mIU/mL (1-11 IU/L)
FSH	4.44 mIU/mL (4.44 IU/L)	2.5-9.1 mIU/mL (2.5-9.1 IU/L)
TSH	1.71 mIU (1.71 IU/L)	0.4-4.5 mIU (0.4-4.5 IU/L)
Prolactin	4.54 ng/mL (4.54 ug/L)	2.64-13.13 ng/mL (2.64-13.13 ug/L)
HCG	< 2 IU/mL (0.2 mIU/mL)	< 5 IU/mL (0.5 mIU/mL)
Creatinine	0.8 mg/dL (70.74 µmol/L)	0.7-1.25 mg/dL (61.9 to 114.9 µmol/L)
Total bilirubin	0.8 mg/dL (13 umol/L)	0.1-1.2 mg/dL (5.13-17 umol/L)
AST	21 U/L (0.35 ukat/L)	17-35 U/L (0.28-0.58 ukat/L)
ALT	29 U/L (0.48 ukat/L)	8-39 U/L (0.13-0.58 ukat/L)
ALP	54 U/L (0.9 ukat/L)	39-113 U/L (0.65-1.88 ukat/L)

Values in parentheses are International System of Units (SI).

Abbreviations: ALP, alkaline phosphatase; ALT, alanine transaminase; AST, aspartate aminotransferase; FSH, follicle-stimulating hormone; HCG, human chorionic gonadotropin; LH, luteinizing hormone; TSH, thyroid-stimulating hormone.

## Treatment

Due to significant discomfort associated with gynecomastia and due to extreme nipple sensitivity to clothing, a shared decision was made to stop TP, and treatment with risedronate 35 mg once weekly was initiated to prevent further decline in BMD.

## Outcome and Follow-Up

Following the discontinuation of TP, the patient reported that his symptoms gradually improved, and the nipple sensitivity and the palpable swelling of the left breast had completely resolved at his follow-up visit 4 months later.

## Discussion

TP, an osteoanabolic agent, is a biosynthetic analog of the 1–34 amino acids of human PTH. TP is FDA-approved for the treatment of postmenopausal osteoporosis with a high fracture risk, treatment of men with primary or hypogonadal osteoporosis, and for treatment of men and women with systemic glucocorticoid-induced osteoporosis (1). It is administered subcutaneously at a daily dosage of 20 μg for 18 to 24 months. Although usually well-tolerated, short-term side effects include nausea, headaches, arthralgias, and dizziness. Alterations in calcium metabolism can be seen rarely, including hypercalcemia, hyperuricemia, and hypercalciuria.

The temporal association between the initiation of TP treatment and the onset of gynecomastia, and improvement of the symptoms and physical findings upon discontinuation of the drug in our patient suggests that TP was the likely cause of gynecomastia in our patient. The effects of TP are mediated through the interaction of G-protein coupled parathyroid hormone receptor (PTHR1) in the cell membrane. Ligand binding to the receptor activates adenylate cyclase, protein kinase A, and protein kinase C. This increases intracellular levels of cAMP and calcium, which in turn increases the number of osteoblasts and bone formation by activation of pre-existing osteoblasts, increased differentiation of lining cells, and reduced osteoblast apoptosis (2).

Parathyroid hormone-related protein (PTHrP) is produced during normal embryonic and postnatal development and acts as a local growth factor in various tissues including bone, cartilage, and mammary gland. In the breast, PTHrP is critical in the formation of mammary mesenchyme, and its effects in the fetus are mediated through PTHR1 (3).

Given that both PTHrP and PTH use the same receptor—PTHR1, we hypothesize that our patient may have had residual embryonic mesenchymal stem cells in his left breast. TP stimulated the proliferation of these residual embryonic mesenchymal stem cells, which may have led to transient gynecomastia in our patient. Staining for PTHR1 on breast biopsy may have confirmed this hypothesis but our patient declined a breast biopsy. He also declined a rechallenge either with TP or with abaloparatide. Abaloparatide also mediates its effects through PTHR1, and it would have been interesting to see if the gynecomastia recurred with abaloparatide.

Although postmarketing reports on TP do mention gynecomastia occurring at a higher frequency in middle-aged males, we did not find any reported case on PubMed search. Our case illustrates the first reported case in the literature of TP-associated gynecomastia in a male with osteoporosis.

## Learning Points

- Teriparatide is an analogue of parathyroid hormone (PTH) used for osteoporosis.
- Parathyroid-related protein (PTHrP) is crucial for embryonic breast, bone, and cartilage formation. PTH and PTHrP exert effects through the same receptor, PTHR1.
- We present a case of unilateral gynecomastia in a male shortly after starting treatment with TP, which resolved within 4 months after discontinuation of therapy. This side effect has not been reported in the literature to date.
- We hypothesize that the likely mechanism for this association may be the presence of residual embryonic mesenchymal stem cells in his left breast with PTHR1 receptors that were stimulated with TP. This postulation needs to be confirmed in future case reports and studies.

## Data Availability

Original data generated and analyzed during this study are included in this published article.

## Abbreviations

BCE: bone collagen equivalents
BMD: bone mineral density
BSAP: bone-specific alkaline phosphatase
GERD: gastroesophageal reflux disease
NTx: collagen cross-linked N-telopeptide
PPI: proton pump inhibitor
PTH: parathyroid hormone
PTHR1: parathyroid hormone type 1 receptor
PTHrP: parathyroid hormone-related protein
TP: teriparatide
